# Targeting the oral plaque microbiome with immobilized anti-biofilm peptides at tooth-restoration interfaces

**DOI:** 10.1371/journal.pone.0235283

**Published:** 2020-07-02

**Authors:** Dina G. Moussa, Conrado Aparicio

**Affiliations:** Department of Restorative Sciences, MDRCBB-Minnesota Dental Research Center for Biomaterials and Biomechanics, School of Dentistry, University of Minnesota, Minneapolis, Minnesota, United States of America; Oregon Health & Science University, UNITED STATES

## Abstract

Recurrent caries, the development of carious lesions at the interface between the restorative material and the tooth structure, is highly prevalent and represents the primary cause for failure of dental restorations. Correspondingly, we exploited the self-assembly and strong antibiofilm activity of amphipathic antimicrobial peptides (AAMPs) to form novel coatings on dentin that aimed to prevent recurrent caries at susceptible cavosurface margins. AAMPs are alternative to traditional antimicrobial agents and antibiotics with the ability to target the complex and heterogeneous organization of microbial communities. Unlike approaches that have focused on using these AAMPs in aqueous solutions for a transient activity, here we assess the effects on microcosm biofilms of a long-acting AAMPs-based antibiofilm coating to protect the tooth-composite interface. Genomewise, we studied the impact of AAMPs coatings on the dental plaque microbial community. We found that non-native all D-amino acids AAMPs coatings induced a marked shift in the plaque community and selectively targeted three primary acidogenic colonizers, including the most common taxa around Class II composite restorations. Accordingly, we investigated the translational potential of our antibiofilm dentin using multiphoton pulsed near infra-red laser for deep bioimaging to assess the impact of AAMPs-coated dentin on plaque biofilms along dentin-composite interfaces. Multiphoton enabled us to record the antibiofilm potency of AAMPs-coated dentin on plaque biofilms throughout exaggeratedly failed interfaces. In conclusion, AAMPs-coatings on dentin showed selective and long-acting antibiofilm activity against three dominant acidogenic colonizers and potential to resist recurrent caries to promote and sustain the interfacial integrity of adhesive-based interfaces.

## Introduction

Dentistry has benefited from the continuous development of esthetic composite restorations that offer both cosmetic and restorative treatments [1] for addressing the most prevalent chronic disease globally, dental caries [2]. However, the durability of composite restorations is notably compromised because of recurrent carious lesions that develop at the margins of the restorations after +3 years [3]. Recurrent caries is the primary reason for failure and replacement of composite restorations [1, 3, 4] as composite restorations are most vulnerable at the adhesive-dentin interface [5], where the restoration is susceptible to biodegradation by water, waterborne agents, and bacteria. Increasing development of bacterial resistance to antibiotics used to treat dental caries [6] and the limited activity of resin-immobilized antimicrobials [7] has raised an urgent need to develop new antibacterial agents that possess significant bacteriostatic activity in the immobilized state.

Antimicrobial peptides (AMPs), also called host defense peptides, are a unique diverse group of small molecular weight proteins with broad spectrum antimicrobial activity against bacteria, viruses, and fungi [8]. The mechanism of action of AMPs to kill microbes varies; however, they usually destabilize biological membranes and form disruptive transmembrane channels [9]. AMPs may also translocate the cell membrane to interact with intracellular targets which are vital to cell survival, such as inhibition of DNA, RNA, certain enzymes, protein folding, and cell wall synthesis [10]. Cationicity and amphipathicity are very important features that determine the selectivity and potency of AMPs [11]. The cationic nature of AMPs has been associated with the AMPs ability to selectively interact with the negatively charged bacterial cell compared to the zwitterionic eukaryotic cells. This selective ionic interactions further enable AMPs to kill bacteria without being significantly toxic to the host cells [12, 13]. Amphipathicity, the feature whereby peptides arrange hydrophilic and hydrophobic amino acid residues along opposing sides of the molecules, imparts AMPs with the ability to partition into the bacterial membrane lipid bilayer causing bacterial disruption [14, 15]. Collectively, AMPs have rapidly captured attention as novel potent therapeutic agents to replace antibiotics [16, 17].

Recently, several trials have implemented AMPs in aqueous solutions to target transiently planktonic bacteria and/or biofilms [16, 18, 19] or incorporating them in dental adhesives [20, 21] to combat oral pathogens via their multimodal mechanisms of action. We have taken a different approach by coating dentin with selected AAMPs having the ability to self-assemble and preferentially bind to hydroxyapatite to create a highly hydrophobic dentin-restoration interface to sustainably resist all waterborne degradative agents [22, 23]. Meanwhile, these selected AAMPs hold potent antibiofilm efficacy to target the complex and heterogeneous oral biofilms where bacteria are unlikely to develop resistance.

We validated our 2-tier protective approach in a previous work [22] where we applied AAMPs coatings on dentin to modulate dentin hydrophobicity and impermeabilize and impart antimicrobial properties. In a subsequent study [23], we sought to test a series of AAMPs to assess their structure-function relationships aiming to select and/or design AAMPs with optimized multifunctional properties for fortifying the otherwise vulnerable adhesive-based interfaces.

Herein, we expanded our understanding of the antibiofilm activity of the best performing AAMPs coatings, as assessed in our previous studies. We conducted a genomic-based study to discern the impact of AAMPs coatings on the plaque biofilm microbial community composition. Moreover, we innovatively assessed the plaque biofilm response to AAMPs coatings *ex-vivo* along an artificially failed dentin-composite interface using advanced nonlinear multiphoton deep bio-imaging, which facilitated interpretation of the translational value of these coatings.

Our 2-tier defensive system (hydrophobic and antibiofilm) is expected to increase the longevity of composite restorations via combating the multifactorial etiology of recurrent caries.

## Methods

### Peptide synthesis

All peptides were synthesized (purity >98%) by AAPPTec (Louisville, KY, USA): **L-GL13K, D-GL13K**, **1018**, **DJK2**, **DJK5, and hLf1-11** (antimicrobial); **L-GL13K-R**, **D-GL13K-R** (controls, non-antimicrobial); and **green fluorescent-labelled L-GL13K** (5-FAM-GKIIKLKASLKLL-NH2) (S1 Table in S1 File).

### Plaque samples collection

After a written informed consent was obtained from all participants, supragingival dental plaque samples from 10 high caries risk subjects were collected from either the occlusal or buccal margin of existing restorations at the University of Minnesota (Institutional Review Board #1403M48865). Each subject was examined for a formal caries risk assessment (CAMBRA) which classifies the caries risk status into 3 categories (low, moderate, high) considering a wide-range of biological predisposing factors and the protective factors, 8 factors each, in addition to the comprehensive clinical examination [24]. All subjects had experienced active carious lesions and were still at high risk for future caries; otherwise, they were in good general health and had not taken antibiotics within 3 months of plaque sampling.

### Selection of the most challenging biofilm

All dental plaque samples were collected using a sterile sickle scaler, and each sample was immediately deposited into a vial containing 1 ml pre-reduced anaerobic transfer medium (Anaerobe Systems, Morgan Hill, CA, USA). Suspension planktonic cultures were grown overnight anaerobically in modified BHI medium (S2 Table in S1 File). Minimum inhibitory concentration (MIC) assay was conducted to screen the susceptibility of all samples to D-GL13K antimicrobial peptide. The bacterial suspension was adjusted to optical density (OD)_600_ = 0.2±0.01 and then diluted 1:20. D-GL13K was dissolved in 0.01% acetic acid and added to sterile 96-well polypropylene microtiter plates at decreasing 2-fold serial dilutions (0–512 μg/ml). Media without peptides were used as controls. Plaque bacteria were inoculated to a final concentration of 5.0×10^5^ CFU/ml per well. The plates were incubated at 37 °C for 24 hours under continuous shaking at 200 rpm. After 24 h of peptide treatment, absorbance at 600 nm was measured using a microtiter plate reader (Bio-Tek Instruments, Winooski, VT, USA). Plaque sample #311 was challenging in the planktonic state and, additionally, it formed the highest adherent biofilm using the Crystal Violet assay as measured by Dr. Robert S. Jones lab (data not shown). Thus, we chose plaque sample #311 for the rest of our experiments as a quite challenging model in both planktonic and biofilm states.

### Formation and characterization of peptides coatings on hydroxyapatite discs

Hydroxyapatite (HA) discs (9.65 mm diameter by 1.52 mm thickness; Clarkson Chromatography Products, Williamsport, PA, USA) were sterilized by autoclaving to be used as plaque biofilm substrates. Both sides of each disc were etched by 32% phosphoric acid gel (Scotchbond^™^ Universal Etchant, 3M, St. Paul, MN, USA) for 15 s followed by 10 s water-rinse and 10 s air-dry. The discs were coated, incubated with a thin layer of peptide solution at 37 °C for 1 min, by 1 mg/ml of either D-GL13K or 1018 or DJK2 followed by 60 s air-dry. The control discs were etched, but were not coated with peptides. All samples were subject to ultrasonication in water bath for 15 min to remove any unattached peptides. X-ray Photoelectron Spectroscopy (XPS; SSX-100, Surface Science Laboratories; Al Kα X-ray, 1 mm spot-size, 35° take-off angle) was used to detect the atomic composition of the coated surfaces.

### Antibiofilm potency of selected AAMPs coatings

Peptide-coated HA discs and controls were inoculated with 200 μl of stock sample from the highly adherent subject # 311 at the aforementioned adjusted inoculum; then 1.8 ml of fresh media was added. The discs were incubated in an anaerobic chamber at 37°C under shaking at 200 rpm for 48 h. The supernatant in each well was gently pipetted out and discs were rinsed with 0.9% NaCl twice to remove the unattached bacteria to perform vitality and viability assays.

#### Live/dead viability assay

Working solution of fluorescent stains (L7012, LIVE/DEAD BacLight Bacterial Viability Kit, ThermoFisher Scientific, Waltham, MA, USA) was prepared by adding 3 μl of SYTO^®^ 9 (live cell stain) and 3 μl of Propidium iodide (PI, dead cell stain) to 1 ml of sterilized Milli-Q^®^ ultrapure water. 100 μl of staining solution was added onto the HA disc. The discs were covered and incubated for 20–30 minutes at room temperature, protected from light. The stained discs were examined by upright fluorescent microscopy using a 40x water immersion lens (Eclipse E800, Nikon, Tokyo, Japan). The excitation/emission maxima for SYTO 9 and PI were 480/500 nm and 490/635 nm, respectively. The peptide coatings were challenged mechanically by ultrasonication in a water bath for 45 min or chemically by immersion in 30% acetic acid (pH = 2.4) for 45 min. Then, the discs were transferred to well plates to regrow the biofilm for additional 5 and 15 days using the same aforementioned protocol. The regrown biofilms were re-assessed using the same Live/Dead assay.

#### Colony-forming unit (CFU) count

As the etched HA substrates were notably rough, biofilms were detached with an optimized method to secure their complete detachment from etched HA substrates. A magnetostrictive ultrasonic scaler (Cavitron^®^ Select SPS ultrasonic scaler type Gen-124, Dentsply, York, PA, USA) was used at medium power without water irrigation. 1.5 ml of cold 0.9% NaCl was added to10 ml glass vials contacting the discs. The openings of the vials were sealed with a plastic paraffin film (Parafilms^®^ AquaPhoenix Scientific Inc. Hanover, PA, USA) around the shaft of the ultrasonic insert (Slimline^®^–FSI-SLI 10S- Dentsply, York, PA, USA). The insert’s tip was immersed in the solution and positioned just above and parallel to the disc surface. The samples were sonicated in non-contact mode for 90 s each. The whole process was done on ice to compensate for the high temperature generated during sonication and thus, cells maintained their vitality. The bacterial cells were collected after additional ultrasonication in water bath for 10 minutes and quick vortexing. One ml was used for DNA extraction and 0.1 ml was used for CFU. For CFU, serial dilutions were prepared, 10^1^ through 10^5−^10^6^ in quadruplicates for each sample to be cultured on Brucella Agar Plates with Hemin and Vitamin K (Teknova, Cat. No B0155, Hollister, CA, USA). The plates were incubated in anaerobic conditions for 24 h and CFU counts were calculated. Live/dead viability assay was conducted on HA discs after biofilm detachment to assess the detachment efficiency using narrow ultrasonic tips for highly porous substrates, such as etched HA compared to conventional biofilm detachment methods.

#### Propidium monoazide (PMA) treatment

PMA treatment was conducted for selective detection of viable bacteria. Prior to DNA extraction, the collected 1ml of bacterial cells was sub-divided into 0.5ml with PMA and the other 0.5ml without PMA treatment for each sample for fulfilling a pairs-matched statistical design (S1 Fig in S1 File). PMA was added to the assigned samples for a final concentration of 50 μM. The samples were incubated in the dark for 10 minutes on a shaker at room temperature. The samples were exposed to a PMA-Lite LED photolysis device light (Biotium, Fremont, CA, USA) to cross-link PMA to DNA for 15 minutes. Cells were pelleted by centrifuging at 5000x g for 10 minutes in preparation for genomic DNA extraction.

#### DNA Isolation protocol and sequencing

Genomic DNA was extracted from all samples; i.e., PMA and non-PMA treated bacteria. The protocol amended from Epicentre MasterPure^™^ DNA Purification Kit and Epicentre MasterPure^™^ Gram Positive DNA Purification Kit was followed (http://homings.forsyth.org/DNA%20Isolation%20Protocol.pdf, last access 03/31/2020).

The extracted DNA was submitted to the University of Minnesota Genomics Center for dual-indexed 16S rRNA gene amplicon sequencing using the V3-V4 region on the Illumina MiSeq platform 300PE.

### Biofilm response to AAMPs coatings along dentin-composite interfaces

#### Ex vivo *artificial gap at the dentin-composite interface*

**Fig 1:**
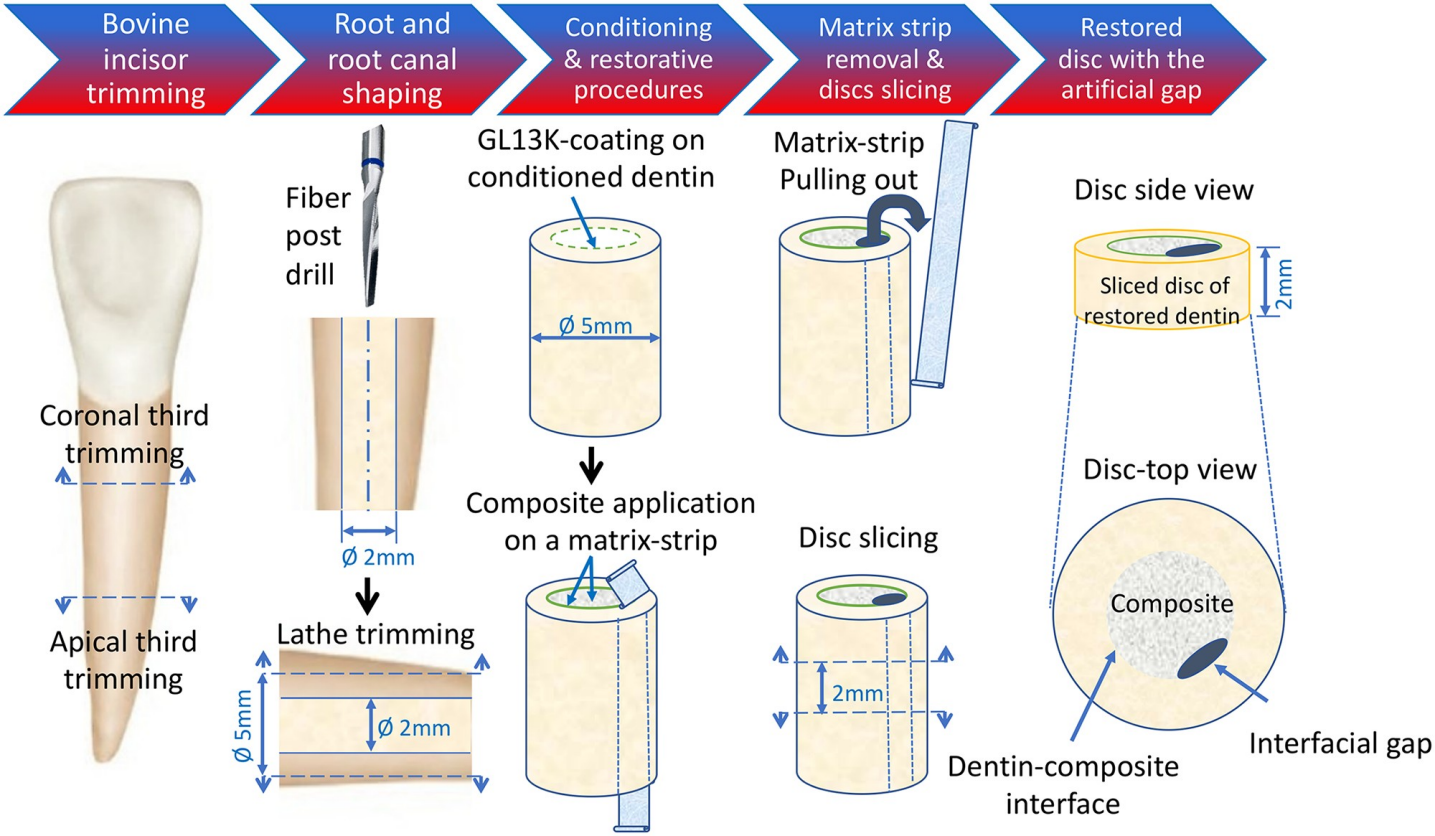
Schematics for the preparation of an *ex vivo* simulated dentin-composite failed interface used to assess the impact of AAMPs-treated interfaces on dental plaque biofilms. A reproducible artificial gap was introduced along the dentin-composite interface of the restored discs using a method adapted from [25]. The radicular dentin tubes were total-etched and filled with dental resin composite (Filtek™ Z250 Universal Restorative 3M, St. Paul, MN, USA) without adhesive. Clear matrix strips (4" x 3/8" x 0.002") (Patterson^®^ Mylar^®^ Matrix Strips, Patterson Dental, Dental Supply, St. Paul, MN, USA) were introduced in the dentin cylinders, against the dentin wall, before composite application. After curing of the composite, the matrix strips were pulled out leaving a gap of ~ 400 μm in thickness along the interface. Then, the restored dentin cylinders were transversally sliced using a pre-calibrated template into 2-mm thick dentin-composite discs. This model was used to assess the biofilm succession along the interface with and without D-GL13K treatment using multiphoton deep bioimaging as detailed in Fig 5.

Roots of bovine teeth were used to prepare restored dentin-composite discs (Fig 1). After the crowns and apical thirds of each tooth were cut off, the root canals were enlarged to 2 mm in diameter, using 1.9-mm diameter fiber post drills (RelyX^*™*^ Fiber Post, 3M, St. Paul, MN, USA). The outer surface of the roots was trimmed down to 5 mm in diameter using a lathe to remove cementum and external layers of dentin. The obtained dentin tubes (5 mm in outer diameter and 2 mm in inner diameter) were water rinsed and stored in 0.5 chloramine T solution at 4 °C until the restorative procedures were performed. The radicular dentin tubes were total-etched for 15 s, water rinsed, air-dried for 10 s each, and filled with dental resin composite (Filtek^*™*^ Z250 Universal Restorative 3M, St. Paul, MN, USA) without adhesive. We introduced an artificial interfacial gap to simulate a failed restored interface by adapting the method by Khvostenko et al. 2015 [25]. Clear matrix strips (4" x 3/8" x 0.002") (Patterson^®^ Mylar^®^ Matrix Strips, Patterson Dental, Dental Supply, St. Paul, MN, USA) were introduced in the dentin cylinders, against the dentin wall, before composite application. After curing, the matrix strips were pulled out leaving a gap of approximately 400 μm in thickness along the interface. Then, the restored dentin cylinders were sliced using a pre-calibrated template into 2-mm thick dentin-composite discs (Fig 1). The restored discs were inoculated as before and incubated in the anaerobic chamber at 37°C under 200 rpm shaking for 9 days and 18 days. Media were refreshed twice a week. The discs were stained using live/dead viability assay using the aforementioned protocol.

#### Multiphoton bioimaging

Multiphoton images were acquired in a Multiphoton upright microscope equipped with an A1R laser scan head (Nikon FN1, Tokyo, Japan). Excitation of the samples was performed with a Spectra-Physics 15W Mai Tai DeepSee tunable IR laser tuned to 870 nm. Non-descanned highly sensitive GaAsP detectors were used to allow visualization of multiple fluorophores located deep within the specimen. The IR laser was operated in pulsed mode and was focused onto the sample using a 25x NA1.1 water-immersion objective with a 2mm working distance suitable for deep imaging. Multiphoton excitation fluorescence was collected in the epi direction in the green (470–550) and orange-red (570–640) channels. The filters used were 425/50, 510/80, and 605/70. The samples were scanned in 512x512 pixel frames (0.14x0.14 μm pixel size) using resonant scanner with a 1 μm step size in Z. Correction for the Z-intensity was activated at 10–12 points/1 mm. Prior Z Drive was selected to control the objective lens movements at a deeper level, up to 2 mm depth, during Z-stack recording. Images were compiled and analyzed with NIS Elements software (Version 4.11.0, Nikon, Tokyo, Japan).

Quantification of red and green cells was done by standard co-localization analysis using Fiji image processing package. The percentage of red cells, which is usually associated to dead cells was calculated as:

$$dead\;cells\;\%=\left(\frac{total\;volume\;of\;red\;cells}{total\;volume\;of\;cells}\right)x100$$

### Statistical analysis

Statistical analysis was performed using R, version 3.3.2 (64-bit) (R Core Team). Means and standard deviations of MICs, CFUs, and percentages of live and dead cell volumes were analyzed by two-tailed two-sample t-test. α = 0.05 was used as cutoff for statistical significance.

#### Sequence data processing and analysis

Illumina data was processed with cutadapt for removal of adapters, DADA2 for quality processing and an amplicon sequence variant table (ASV) analysis following current suggested practices. Primers where removed with FilterandTrim, paired reads were merged with mergePairs, Bimeras where removed in with removeBimeraDenovo, and Silva version 132 was used for taxonomy identification. Sequences with a length < 251 and > 255 were removed as well as sequences with a total read count across all samples equal to 1. Further analysis of the ASV table was performed in R with the vegan, reshape2, ggplot2, grid, gridExtra, plyr, dplyr packages. The alpha diversity indices of Shannon, Simpson, Inverse Simpson (InvSympson) were calculated at 97% identity. Beta diversity analysis was performed by principal coordinates analysis (PCoA) based on Bray-Curtis distances at the Operational Taxonomic Unit (OTU) level. Using R-vegan function Adonis, Permutational multivariate analysis of variance (PERMANOVA) and TukeyHSD for multiple comparisons were used on treatment variables and alpha diversity statistics Shannon, Simpson and InvSympson [26, 27]. Differences were considered significant when p < 0.05.

## Results

### Antimicrobial potency of AAMPs

**Fig 2:**
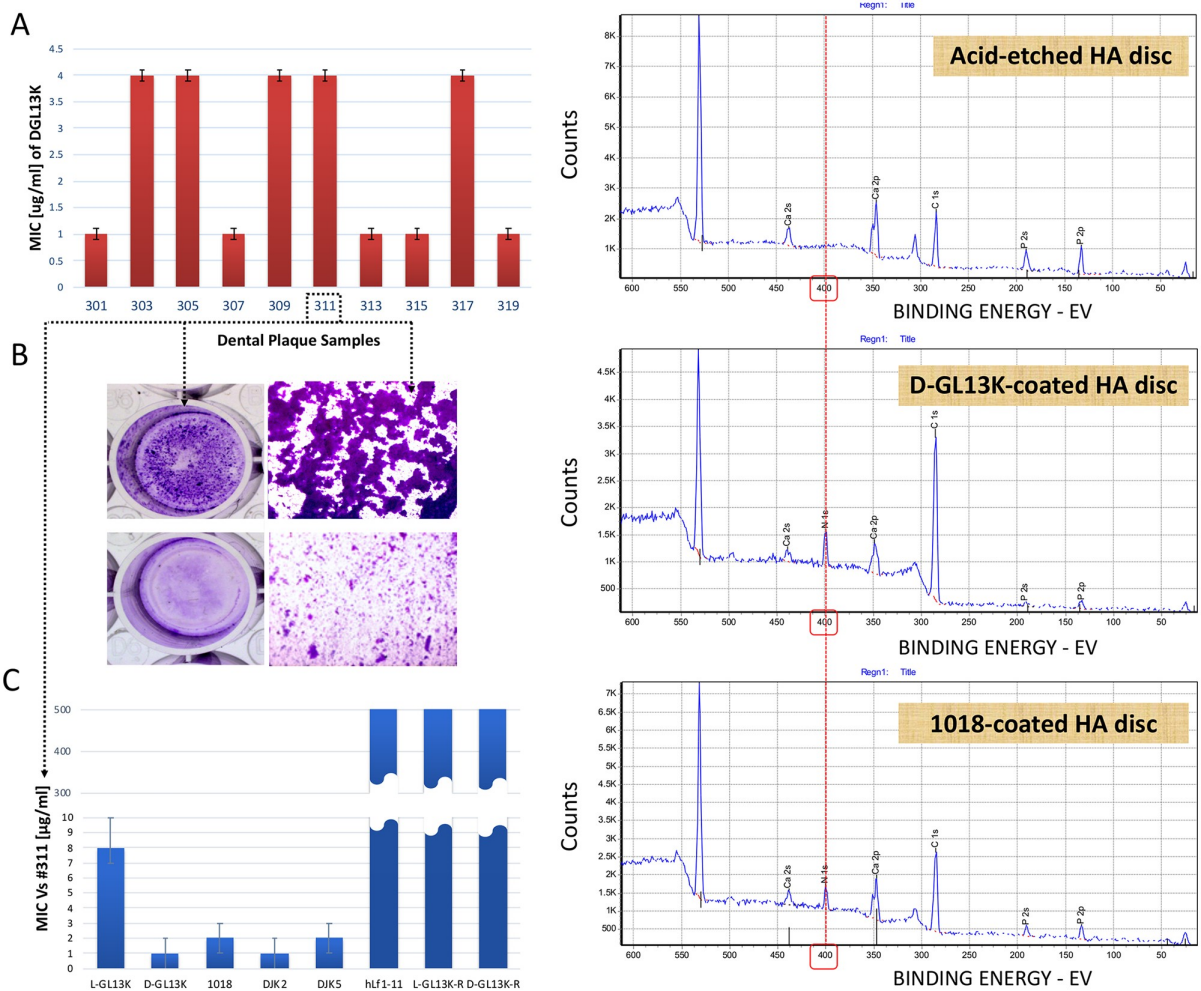
Antimicrobial effect of peptide candidates on dental plaque communities. A) The minimum inhibitory concentrations (MICs) of D-GL13K against 10 different supragingival plaque samples collected from caries active individuals. Bars show average ± standard deviation (n = 3). B) 6-day-old highly adherent plaque biofilm of #311 sample (top panels). Bottom panels show #311 sample treated with 25μg/ml D-GL13K [22]. C) MICs of antimicrobial and non-antimicrobial control peptides against #311 plaque sample [23]. Bars show average ± standard deviation (n = 3). D) X-ray photoelectron spectroscopy (XPS) spectra for peptide coatings on HA discs. Top panel shows the surface atomic composition of acid-etched (32% H_3_PO_4_) control HA discs. Middle and bottom panels show the surface atomic composition of etched HA discs after coating with D-GL13K (an all D-amino acids peptide) and 1018 (an all L-amino acids peptide), respectively. The red dashed line shows the position of the nitrogen-N1s peak. The appearance of the nitrogen peak in peptide-coated HA discs is indicative of the presence of the peptides on these discs. HA: Hydroxyapatite.

MICs of D-GL13K against cultures of all 10 plaque biofilm samples were in the range 1–4 μg/ml (Fig 2A). The high adherence of sample #311 was validated (Fig 2B) and D-GL13K significantly inhibited #311 plaque biofilm (around 4-fold) at a concentration as low as 25 μg/ml (Fig 2B). Potency of the rest of AAMPs candidates was tested against cultures of the highly adherent #311 sample. Several batches of all D-amino acid peptides (D-GL13K, DJK2, and DJK5) and 1018 showed the lowest MICs values (MIC = 1–2 μg/ml) followed by L-GL13K (MIC = 8 μg/ml). The controls, scrambled amino acid versions of D- and L-GL13K peptides did not show inhibitory effect up to 512 μg/ml (Fig 2C). We later assessed that DJK5 is cytotoxic [23] and thus, DJK5 was excluded from subsequent experiments. Accordingly and for the rest of our experiments, we narrowed the focus to the best performing AAMPs (D-GL13K, DJK2, and 1018) as they combined high antimicrobial potency, amphipathicity, and cytocompatibility. Selected AAMPs, 1018 (L-amino acids AAMP) and D-GL13K (D-amino acids AAMP), were immobilized on HA discs and the AAMPs coatings were characterized by XPS versus the controls (32% H_3_PO_4_ etched HA discs) (Fig 2D). The appearance of the Nitrogen-N1s elemental peak in the XPS spectra of AAMPs coated HA discs confirmed the formation of the AAMPs coatings.

### Antibiofilm potency of selected AAMPs coatings

**Fig 3:**
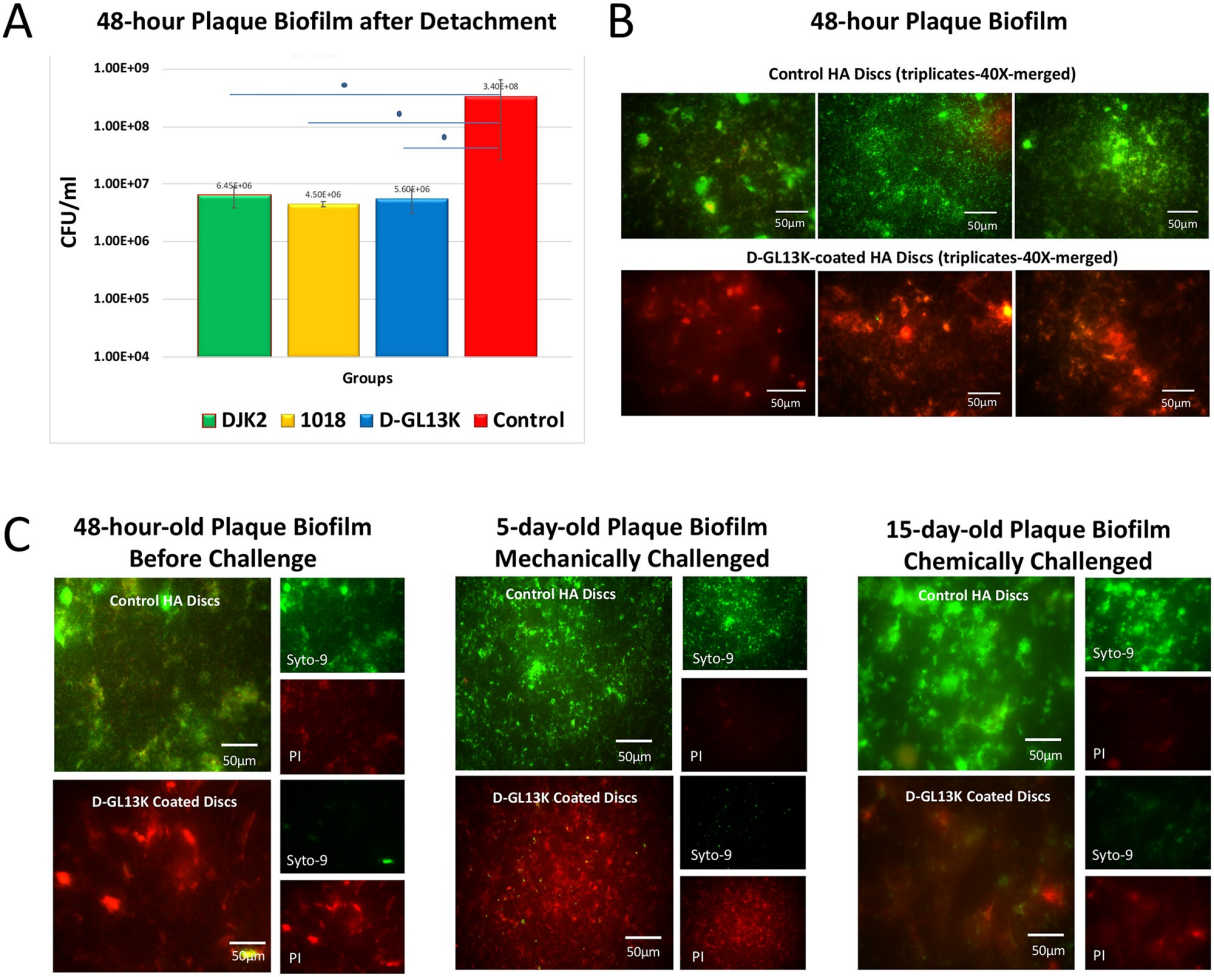
Antibiofilm effect of D-GL13K, DJK2, and 1018 coated HA discs on dental plaque biofilms. A) Cell death quantification by counting colony forming units (CFU) for detached bacteria biofilms from HA discs coated with all AAMPs. Control group is 32% phosphoric acid-etched non-coated HA discs. N = 3. B) Merged live (green) and dead (red) bacteria viability assay images of 48-hour plaque biofilms grown on HA discs with (bottom image) and without (top image) D-GL13K coatings. C) Live and dead viability assay images of 48-hour (left), 5-day (middle), and 15-day-old (right) plaque biofilms grown on HA discs with (bottom images) and without (top images) D-GL13K coating. Middle panel shows regrown biofilms for 5 days after 45 min of ultrasonication (mechanically challenged D-GL13K coating). Right panel shows regrown biofilms for 15 days after 45 min immersion in 30% acetic acid (chemically challenged D-GL13K coating). Merged images are on the left side of each group and separated live (top)/dead (bottom) bacteria images are on the right side of each group.

CFUs for 48-h-biofilm detached from all AAMPs-coated HA discs showed a statistically significant reduction of biofilm of more than 1.5 log (10) compared to non-coated controls (32% phosphoric acid-etched HA discs) (Fig 3A). An almost complete detachment of grown biofilms on HA discs was achieved using magnetostrictive ultrasonic inserts for efficiently detaching biofilms from porous substrates (S2 Fig in S1 File).

While the live/dead assay for control biofilms showed well-organized network of bacteria structures, D-GL13K coatings led to uniform and marked loss of the integrity of bacterial membranes that is usually associated with bacterial cell death across the HA discs after 48h hours of biofilm growth (Fig 3B). The chemically- and mechanically-challenged D-GL13K coatings, immersed in 30% acetic acid or ultrasonicated respectively for 45 min, had sustained antibiofilm activity against 5-day and 15-day regrown biofilms, respectively (Fig 3C).

### Genomic-based analysis for the effects of AAMPs coatings on plaque biofilm communities

**Fig 4:**
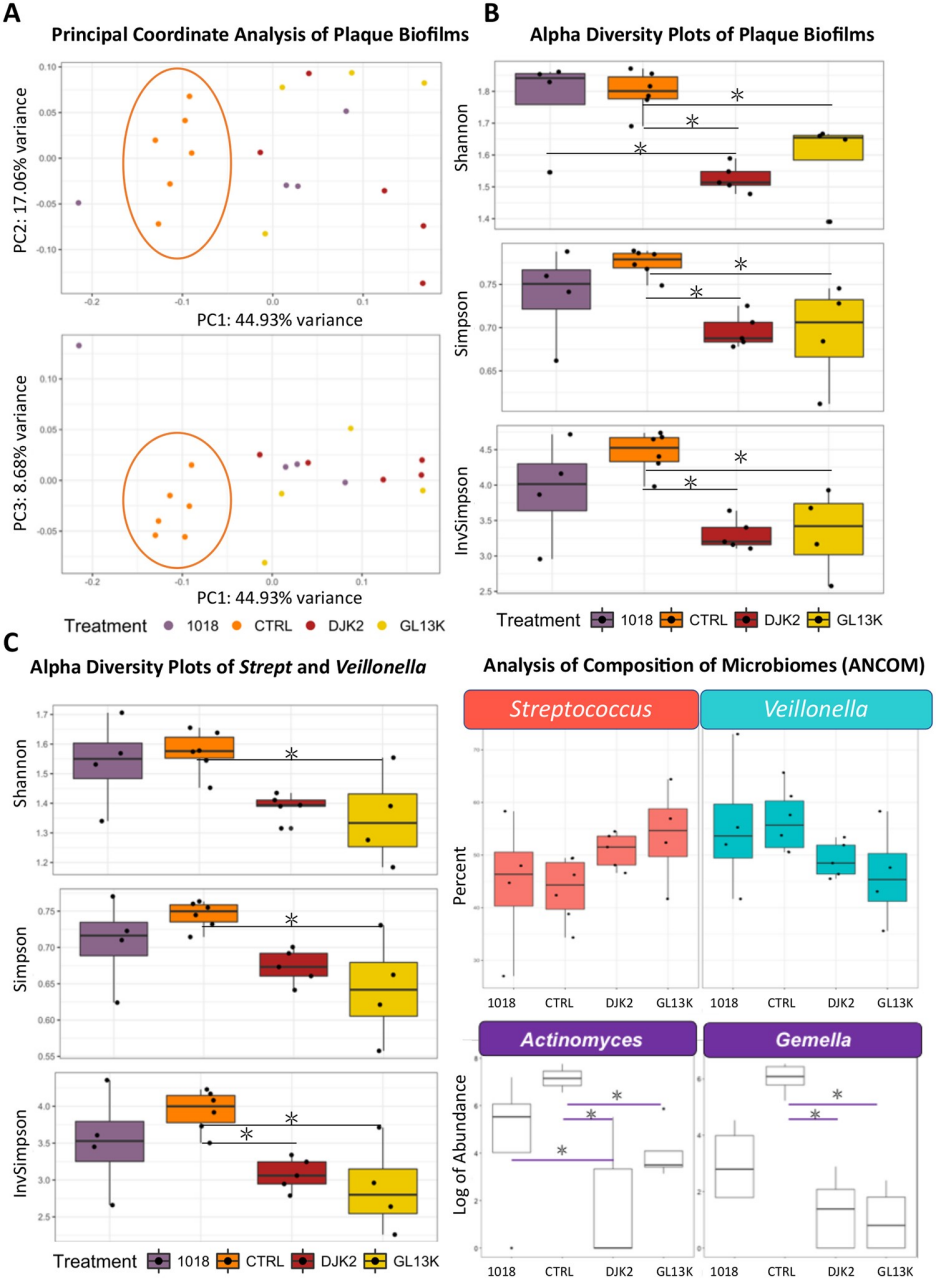
Effect of D-GL13K, DJK2, and 1018 coated HA discs on the dental plaque biofilms microbial community composition using 16s ribosomal (rRNA) gene next generation sequencing. A) Principal coordinates plots shows the distance and relatedness between taxonomic data of tested populations. B) Alpha diversity plots show the genus-level change in bacterial richness (number of bacterial taxa) and bacterial evenness (relative abundance of bacterial taxa). Shannon index (top panel) is sensitive to bacterial richness, Simpson index (middle panel) is sensitive to bacterial evenness, InvSimpson (bottom panel) is the inverse of the Simpson index diversity estimator. C) Alpha diversity plots show the change in bacterial richness and evenness of highly abundant *Streptococcus* and *Veillonella* combined. D) Analysis of composition of microbiomes (ANCOM) statistical framework shows the bacterial selectivity of tested AAMPs for *Streptococcus*, *Veilonella*, *Actinomyces* and *Gemella*.

Principal Coordinate Analysis (PCoA) shows the distance between plaque biofilm samples in multidimensional space (Fig 4A). There was a marked distinction between plaque biofilm grown on control samples (cluster encircled in orange ring) and on AAMPs-coated samples along the coordinate PC1, which accounted for the largest proportion of total variance.

Bacterial diversity; i.e., richness and evenness, was assessed for various measures of alpha diversity (diversity within a sample). Shannon index, accounts for both abundance and evenness, showed that all D-amino acid AAMPs coatings (D-GL13K and DJK2) significantly reduced the bacterial community richness compared to controls (p-value = 0.02 and 0.002, respectively). Sensitive indices for community evenness showed similar significant differences (Simpson index: p-value = 0.02, 0.018; and InvSimpson index: p-value = 0.01, 0.0049 for DGL13K and DJK2, respectively vs controls). Unlike D-peptides coatings, coatings of the L-peptide 1018 did not have significantly different bacterial richness and evenness compared to controls (Fig 4B).

We studied the plaque biofilm bacterial selectivity in the uneven communities on D-peptides coatings. We found the breadth of bacterial diversity embraced six genera and was dominated by *Veillonella* and *Streptococcus* for all groups (85% of the total sequences) (S3 Table and S3 Fig in S1 File). Thus, we analyzed the highly abundant taxa separately using the same aforementioned diversity indices. D-GL13K coatings induced a significant reduction in the number and relative abundance for both genera combined versus controls (p-value = 0.02, 0.01, 0.01 for Shannon, Simpson and InvSimpson, respectively). DJK2 coatings induced similar effects to D-GL13K coatings versus controls (p-value = 0.05, 0.08, 0.03 for Shannon, Simpson and InvSimpson, respectively). All diversity parameters for plaque biofilm grown on 1018 coatings were consistently similar to controls, but did not reach statistical significance compared to D-peptides (Fig 4C). We used the analysis of composition of microbiomes (ANCOM) index to monitor *Veillonella* and *Streptococcus* behavior separately for the different groups. ANCOM confirmed that *Veillonella* was specifically targeted by D-peptides (Fig 4D-upper panel).

For the less abundant taxa, ANCOM showed that D-peptides coatings significantly reduced *Actinomyces* and *Gemella* (n = 6, p-value = 0.002 and 0.034, respectively) (Fig 4D-lower panel). This finding was confirmed by the indicator value (IndVal) index that measures the association between bacterial taxa and a site group [28] where *Actinomyces and Gemella* were significantly associated with controls (p-values = 0.001 and 0.004, respectively).

### Biofilm response to AAMPs coatings along dentin-composite interfaces

**Fig 5:**
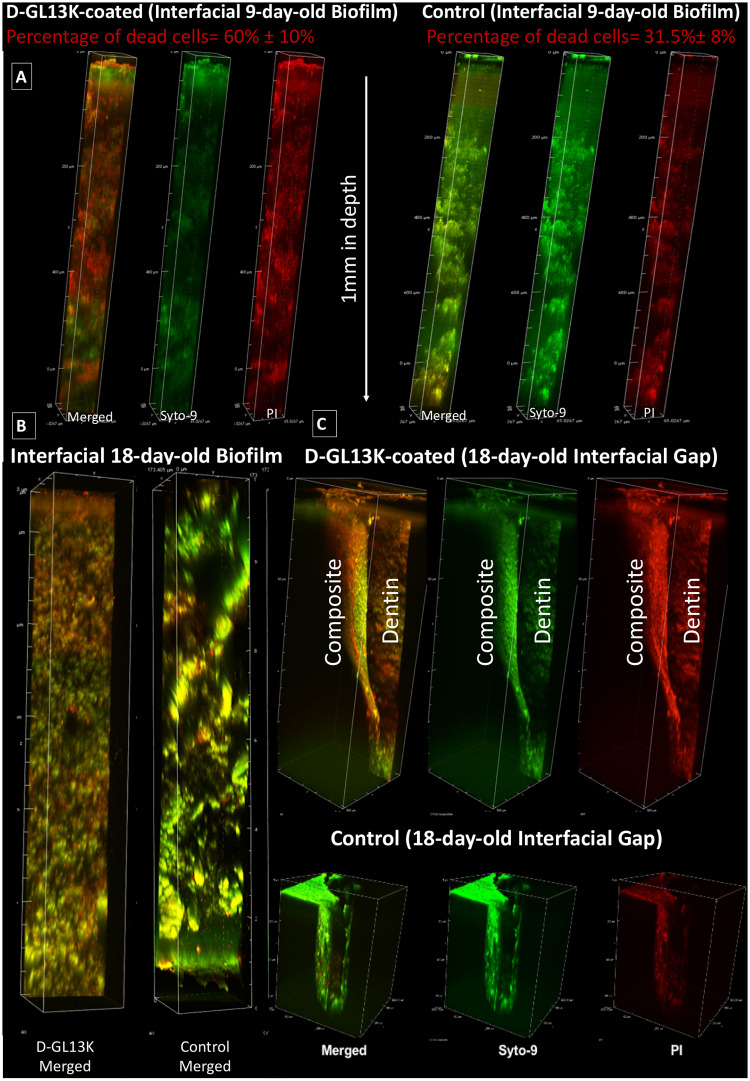
Multiphoton fluorescence 3D rendering of plaque biofilms along dentin-composite interfacial artificial gaps up to 1 mm in depth created as shown in Fig 1. A) 9-day-old plaque biofilm and B) 18-day-old plaque biofilm along D-GL13K treated (left) and control non-peptide treated (right) interfaces. Quantified dead cells in A) are average percentage values ± standard deviations. C) Zoomed-out images of the 18-day-old plaque biofilm grown at the interfacial gap between dentin and composite restorations. Top panel shows the plaque biofilm response to D-GL13K coated dentin and composite restoration; that is the straight surface and the curved surface of the artificial gap, respectively. Bottom panel shows a control non-coated sample. In A) and C), left channels (red+green = merged live and dead bacteria), central channels (Syto-9, green = live bacteria), and right channels (PI, red = dead bacteria).

D-GL13K-coated dentin significantly increased the percentage of dead bacteria in plaque biofilms grown for 9 days along the dentin-composite interfacial gap promoted by the multiphoton deep bioimaging compared to controls, p-value = 0.018 (Fig 5A, S4 Fig in S1 File, S1 and S2 Videos). D-GL13K coatings maintained their antibiofilm potency for long periods as visualized in the 3D rendering of the18-day-old plaque biofilm bounded by the dentin and composite sides of the interface (Fig 5B and 5C, S3 and S4 Videos).

## Discussion

The caries process is initiated by dental plaque [29] that encompasses diverse bacteria in the biofilm state. This biofilm is considered one of the most complex biofilm systems in nature [30]. Plaque biofilm accumulation on restorations is associated with the development of recurrent caries at the vulnerable dentin-composite interface [31]. Accordingly, there is a critical need for preventing dental plaque succession and targeting the microorganisms responsible for recurrent caries, with particular relevance for composite restorations as they are prone to accumulate thick biofilms [32, 33].

We developed a long-acting antibiofilm and hydrophobic dentin coating technology using potent and cytocompatible AAMPs to resist recurrent caries at dentin-composite interfaces [22] and here, our main goal was to determine the effects of the AAMP coatings on the microbiome of oral biofilms and their effectiveness at *ex vivo* dentin-composite interfaces. Noteworthy, we studied the physico-chemical prosperities of all the aforementioned peptides in a preceding manuscript [23].

D-GL13K exhibited high antimicrobial efficacy at very low concentrations against multiple dental plaque cultures collected from cariogenic subjects (Fig 2A). However, biofilms show increased resistance to antimicrobial agents than suspension cultures [34]. Still, D-GL13K successfully inhibited the formation of highly adherent plaque biofilms by suppressing >70% of biofilm biovolume compared to controls (Fig 2B).

While some antimicrobials are known to lose their activity after immobilization [35], we have previously shown that L-GL13K peptides retained activity after covalent immobilization on titanium [36] and after electrostatic adsorption on etched HA discs even after extensive washing and ultrasonication [22]. This property is shared with other antimicrobial peptides [37]. Likewise, D-GL13K coatings on HA discs effectively triggered bacterial cell death in plaque biofilms compared to controls (Fig 3B). We challenged the coatings mechanically and chemically to trigger their detachment and/or degradation; however, persistent and strong antimicrobial efficacy was displayed after the challenge and for long periods of plaque biofilm culturing (Fig 3C). Although these AAMPs are immobilized through non-covalent interactions with HA discs (Fig 2D) or dentin [23], we previously demonstrated that there was no detectable release of these AAMPs coatings when exposed to oral simulative fluids [23]. The combined electrostatic and hydrophilic interactions between polar/positively-charged amino acids of peptides and the negatively-charged hydrophilic etched-mineral phase might account for the robust stability of D-GL13K coatings and thus, their sustained antibiofilm efficacy. The immobilization patterns of our peptide coatings on the hydroxyapatite mineral phase are detailed in a previous work [23].

Aiming to increase the efficiency and versatility of our AAMPs-based technology, we tested a series of coatings with alternative AAMPs. While GL13K presumably kill bacteria by partial micellization followed by membrane rupture [38]; 1018 and DJK2 kill bacteria by preventing the intracellular accumulation of guanosine penta- and tetra-phosphates [16]. The diverse bactericidal mechanisms of these peptides motivated our interest in assessing their effects on the dental plaque biofilm microbial community composition; specifically on first, the breadth of bacterial diversity; second, the antimicrobial effect of the cytocompatible AAMPs coatings; and third, the bacterial selectivity of the tested AAMPs coatings.

*Streptococcus and Veillonella* were dominating in our plaque biofilms (S3 Table and S3 Fig in S1 File). Even though the carious origin of collected plaques may explain the reduced diversity as dysbiotic communities tend to be less diverse [39, 40], we cannot rule out that some species may have been lost during *in vitro* culturing with modified BHI (S2 Table in S1 File). An alternative medium that may support the growth of a wider range of species has been proposed to maintain bacterial diversity approaching that of the human oral microbiome [40, 41]. However, the use of this alternative medium did not appear to increase the diversity in our system [42]. Also, different substrates, e.g.; teeth enamel, HA and dental composites, might affect the biofilm composition [43]. However, we had previously compared biofilm composition grown on HA versus composite resin discs and found no notable differences between these biofilms [42]. In fact, one cannot rule out that differences of biofilms on different substrates might be dependent on specific testing conditions.

PCoA plots displayed the induced taxonomic shift for AAMPs-coated samples vs control samples (Fig 4A). By dissecting the diversity components, we found that D-peptides coatings induced significantly uneven plaque biofilm communities compared to controls but 1018-coatings had bacterial evenness similar to that of controls (Fig 4B). Evenness accounts for the relative abundance of various bacterial taxa and it increases as taxa are more evenly distributed. Thus, the diversity indices inferred that D-peptides not only were more potent but also they selectively targeted specific taxa compared to the L-peptide and controls. Nonetheless, validation of this bacterial selectivity needs to be further addressed in the presence of salivary pellicle, which might influence the attachment of early colonizers [44]. The high resistance of the non-native D-peptides to proteases-induced digestion and degradation might be responsible for their robust performance [16, 45]. We have also recently demonstrated that the differences between D- and L-peptides in the dynamics of peptide structural arrangement/assembly is correlated with their antimicrobial potency [46]. Understanding of the impact of these AAMPs on different commensal and pathogenic bacteria is crucial for potential applicability, thus we further study the bacterial selectivity of our AAMPs coatings.

We analyzed the bacterial selectivity in two increments. The first increment analyzed the highly abundant taxa using the same diversity indices used for the whole plaque community (Fig 4C). The second increment determined the less abundant taxa using ANCOM index for comparing the composition of microbiomes [47] (Fig 4D) and IndVal index for measuring the association between bacterial taxa and tested groups [28].

The diversity indices showed a consistent decrease in richness and relative abundance of *Streptococcus* and *Veillonella* combined on D-GL13K and DJK2 coatings compared to controls. However, once again, 1018 coatings did not provoke these same effects. By separating *Streptococcus* from *Veillonella*, ANCOM index confirmed the diversity indices for *Veillonella* as D-peptides coatings showed a tendency to target *Veillonella* compared to 1018 coatings and controls. Targeting *Veillonella* could have a strong impact on resisting recurrent caries as it is the most common taxa in recurrent caries biofilms around Class II composite restorations [48]. Furthermore, it has a central role for species succession in developing dental plaque [49]. The binary relation in the combined comparison of *Veillonella and Streptococcus* may explain the consequent increase of *Streptococcus* for the same groups. Since our sequencing resolution was at the genus-level, we were unable to determine species selectivity within these genera that entail a range of pathogenic and commensal bacteria. Thus, further analyses at the species-level with a larger sample size are required to detect the precise impact of these peptides on the related species/strains of *Veillonella and Streptococcus*.

D-GL13K and DJK2 coatings induced a significant reduction of the less abundant taxa, *Actinomyces* and *Gemella*, compared to controls and 1018 coatings (Fig 4D-lower panel). Observably, both genera are acidogenic early colonizers. Acidogenic bacteria produce acids as a byproduct of carbohydrate metabolism, which results in demineralization of tissues and initiation of caries [50]. *Actinomyces* is one of the three dominant genera within the first 4h of colonization of tooth surfaces [51]. These targeted genera were relatively less abundant, but collectively they can have a strong impact on biofilm succession and acid-induced dysbiosis. Besides, the suppression of the acidogenic *Actinomyces* and *Gemella* might have indirectly contributed to the aforementioned specific targeting of *Veillonella* by the D-peptides since growth of *Veillonella* is dependent on acidic secondary byproducts produced by other microorganisms [52, 53].

The L-peptide 1018 showed an induced shift in the complex microbial communities (Fig 4A), e.g., affected the abundance of the culturable *Actinomyces* and the non-culturable *Gemella* (Fig 4D). However, the overall targeting behavior of 1018 was not significant since it minimally impacted the highly abundant *streptococcus* and *Veillonella* (Fig 4C). That was confirmed by the Shannon diversity index, which was almost the same for 1018 and controls where slight differences between these groups were observed in the more sensitive indices to bacterial evenness, Simpson and InvSimpson (Fig 4B).

We implemented PMA treatment for selective quantification of viable bacteria via suppressing the dead cells. PMA-treated samples clustered differently than non-PMA treated samples on PC2 and consistently showed less dispersed behavior compered to non-PMA treated samples (S5 Fig in S1 File). However, none of the diversity indices was statistical significant different between PMA and non-PMA treated samples (S5 Fig in S1 File). Our relatively small sample size might account for the lack of statistical significance.

The impact of the previous findings was assessed by advanced multiphoton bioimaging of plaque biofilm throughout simulated dentin-composite failed-interfaces. We chose the multiphoton nonlinear optics to achieve deep imaging [54], which is inaccessible to 1-photon confocal microscopy [55]. Additionally, multiphoton strongly suppresses the background signal, markedly reduces phototoxicity to the focal region, and supports optical sectioning for high-resolution imaging [56, 57]. 3D renderings of the biofilms proved that D-GL13K-coated dentin significantly triggered bacterial cell death in plaque biofilm grown up to 18 days along the interface and extended over 1 mm in depth (Fig 5A and 5B, S1 and S2 Videos). Interestingly, the bactericidal activity was intense on the D-GL13K-coated dentin side of the gap rather than on the uncoated composite side, especially at the distant part of the curved surface of the composite restoration (Fig 5C, S4 Video). Although the antibiofilm efficacy of these immobilized peptides was mainly constrained to the proximity of the coating, continuous shaking motion might have contributed to extend the antibiofilm effect of AAMPs-coatings to close areas of the non-antimicrobial composite side (S4 Video) and interfered with the completion of the biofilm life-cycle (S6 Fig in S1 File). Notably, 18-day biofilm weren’t enough to mimic recurrent caries induction; however, after the samples were exposed to thermo-mechanical challenges simulating 1-year of clinical use, the physico-chemical efficacy of the peptide coatings had endured [23]. Besides, the artificial gaps we created were markedly wider than the interfacial micro-gaps seen in clinics which posed a notable challenge to the AAMPs coatings. Still, experiments with biofilms older than 18-day are needed to further support the potential clinical benefits of this AAMPs-based technology for resisting recurrent caries.

## Conclusion

Coatings on dentin with non-native cytocompatible AAMPs showed a selective antimicrobial potency against two crucial acidogenic initial colonizers as well as the most highly abundant taxa associated with failed composite restorations. Our *ex-vivo* studies demonstrated the significant impact of AAMPs coatings on the biofilm succession along dentin-composite interfaces showing a potential to resist recurrent caries in clinical settings.

## Supporting information

S1 File (DOCX)

S1 Video Multiphoton fluorescence 3D rendering of 9-day-old plaque biofilm along the dentin-composite interface up to 1 mm in depth (non-coated control). Right channel (PI red = dead bacteria); central channel (Syto-9 green = live bacteria); left channel (red+green = merged live and dead bacteria).
(WMV)

S2 Video Multiphoton fluorescence 3D rendering of 9-day-old plaque biofilm along the dentin-composite interface up to 1 mm in depth (D-GL13-K treated). Right channel (PI red = dead bacteria); central channel (Syto-9 green = live bacteria); left channel (red+green = merged live and dead bacteria).
(WMV)

S3 Video Multiphoton fluorescence 3D rendering of the 18-day-old plaque biofilm at the interfacial gap between dentin and composite restoration (non-coated control). Right channel (PI red = dead bacteria); central channel (Syto-9 green = live bacteria); left channel (red+green = merged live and dead bacteria).
(WMV)

S4 Video Multiphoton fluorescence 3D rendering of the 18-day-old plaque biofilm at the interfacial gap between dentin (straight surface) and composite (curved surface) restoration (D-GL13K-treated). Right channel (PI red = dead bacteria); central channel (Syto-9 green = live bacteria); left channel (red+green = merged live and dead bacteria).
(WMV)
