# Supplementary material for: Targeting the oral plaque microbiome with immobilized anti-biofilm peptides at tooth-restoration interfaces
Source: PLoS One. 2020 Jul 2;15(7):e0235283. doi: 10.1371/journal.pone.0235283 (PMC7331992; doi:10.1371/journal.pone.0235283)
Supplement: S1 File — (DOCX) [file pone.0235283.s001.docx]

## Supporting Information

**Targeting the Oral Plaque Microbiome with Immobilized Anti-biofilm Peptides at Tooth-restoration Interfaces**

**Dina G. Moussa^1#^ and Conrado Aparicio^1*^**

**^1^MDRCBB-Minnesota Dental Research Center for Biomaterials and Biomechanics, Department of Restorative Sciences, School of Dentistry, University of Minnesota, Minneapolis, Minnesota, U.S.A.**

***Corresponding author: Conrado Aparicio, PhD. Professor; University of Minnesota; 16-250A Moos Tower, 515 Delaware St. SE, Minneapolis, MN 55455, USA;** [apari003@umn.edu](mailto:apari003@umn.edu)

**^#^ Current Affiliation: College of Dentistry, University of Saskatchewan, 105 Wiggins Rd, Saskatoon, Saskatchewan S7N 5E4 , Canada**

**S1 Table. Tested peptides and their molecular properties.** Uppercase letters denote L-amino acids and lowercase letters denote the D-amino acids in the sequences of the peptides. Molecular properties obtained from Biofilm-Active AMPs database (<http://www.baamps.it/>, last accessed 03/31/2020). References in table [1-6]

**S2 Table. Composition of Modified Brain Heart Infusion (BHI) medium** (RM188, HiMedia, West Chester, PA, USA)**.**

**S1 Fig. Experimental design diagram for the genomic DNA analysis of plaque biofilms grown on AAMPs-coated HA discs for 48 hours.** Plaque samples were collected from caries-active subjects. The controls are etched-HA discs with 32% phosphoric acid gel. The tested groups are etched and 1mg/ml AAMPs-coated HA discs. AAMPs were D-GL13K, DJK2 (all D-amino acids peptides); and 1018 (all L-amino acids peptide). Each group had 3 replicates which were subdivided into PMA treatment and non-PMA treatment, 3 samples each in pairs-matched design. AAMPs: amphipathic antimicrobial peptides; HA: hydroxyapatite; PMA: propidium monoazide.

**S2 Fig. Impact of biofilm detachment methods on plaque biofilms grown on HA discs.** Merged images of live/dead viability assay for remaining plaque biofilms on HA discs after detachment of biofilms grown for 48h. Remaining biofilm after 15 minutes sonication in ultrasonic bath (top) or using a narrow tip with ultrasonic generator (bottom).The mode of transmission of the ultrasound energy to the sample could explain the more effective detachment of biofilms from the HA disc surfaces using the ultrasonic tip compared to the traditional ultrasonic water bath. Using the narrow ultrasonic tip, the transmitted energy is delivered all directly to the surface of the discs in the form of microstreaming and shock waves minimizing the energy losses. However, using an ultrasonic bath, the ultrasound energy in the form of small vacuum bubbles is lost when transmitted through both water and glass vial [7].

**S3 Table. Detected genera assignments on the taxonomic hierarchy in plaque biofilms using 16s rRNA gene next generation sequencing.** *Streptococcus* and *Veillonella* were highly abundant (85% of the total sequences).

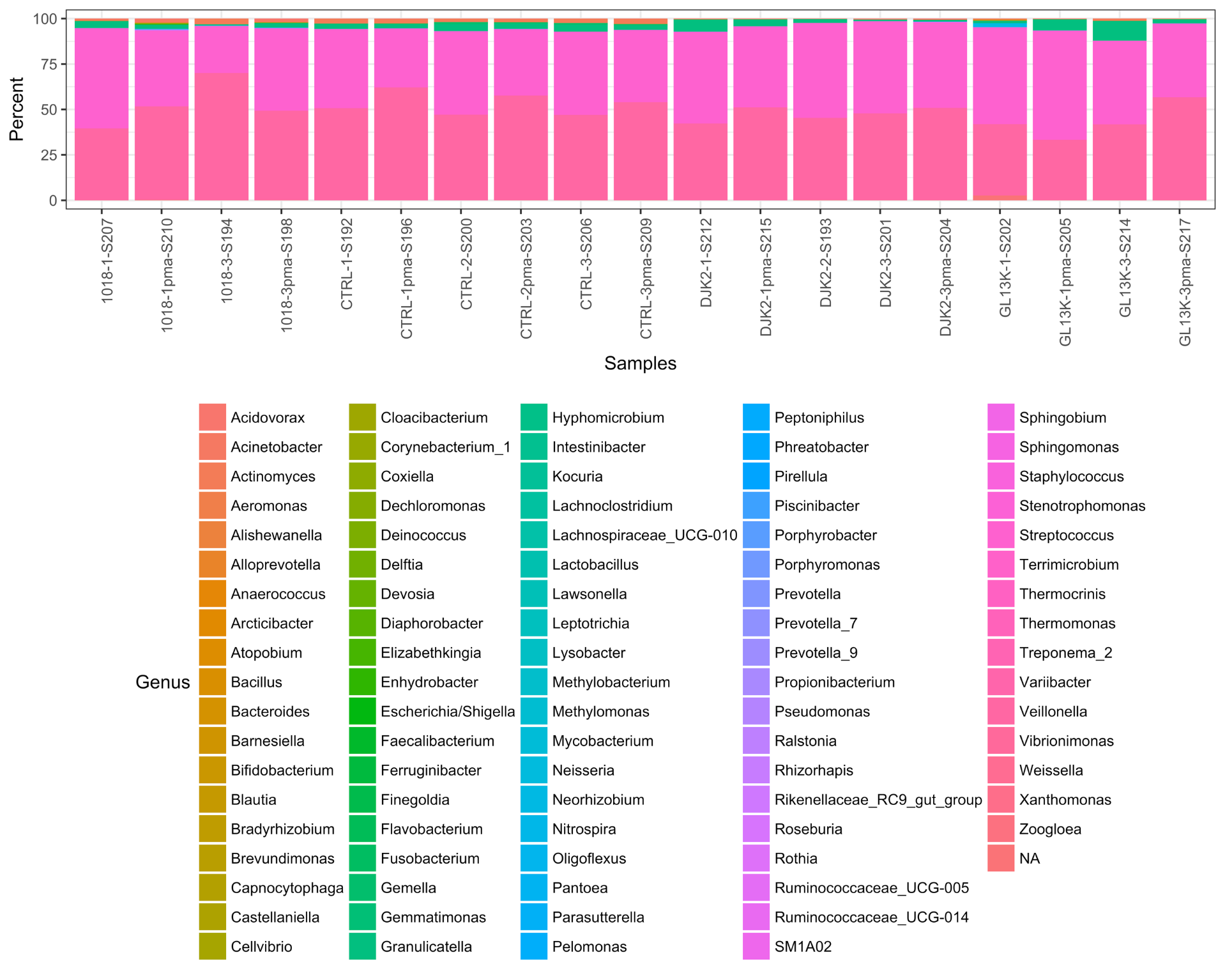


**S3 Fig. Stacked bar graph showing genus level taxonomic composition for each individual sample, expressed as a proportion of reads.** Each sample ID expressed twice on the x-axis with or without “PMA” denoting the split-half samples with or without the pre-amplification treatment for selective quantification of viable bacteria.

**S4 Fig. Percentage of dead cells in the 9-day-old plaque biofilm at the dentin-composite interface.** Values are average ± standard deviation, n=6. * indicates statistical difference between groups (p-value=0.018).

**
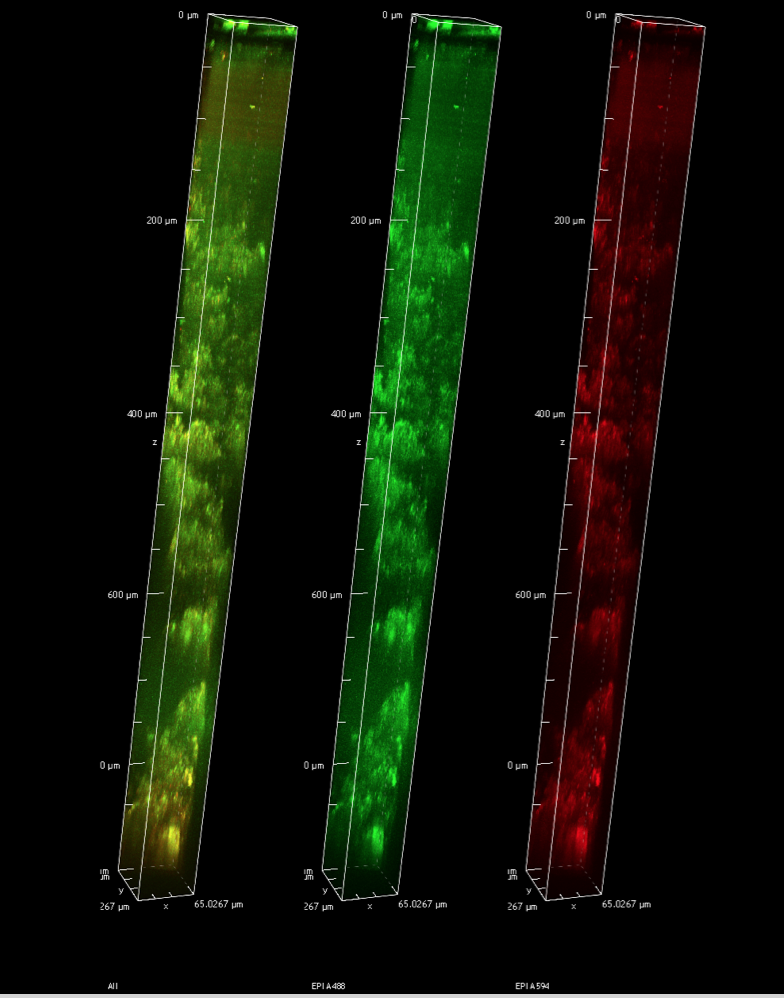
**

**Merged**

**Syto-9**

**PI**

**PI**

**Syto-9**


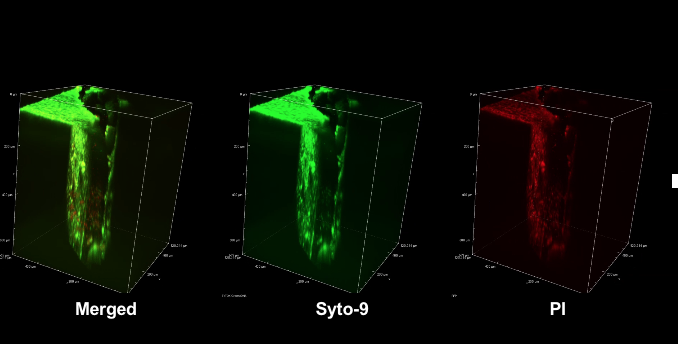


Control (18-day-old Interfacial Gap)

**S1 Video. Multiphoton fluorescence 3D rendering of 9-day-old plaque biofilm along the dentin-composite interface up to 1 mm in depth (non-coated control).** Right channel (PI red=dead bacteria); central channel (Syto-9 green=live bacteria); left channel (red+green=merged live and dead bacteria).

**
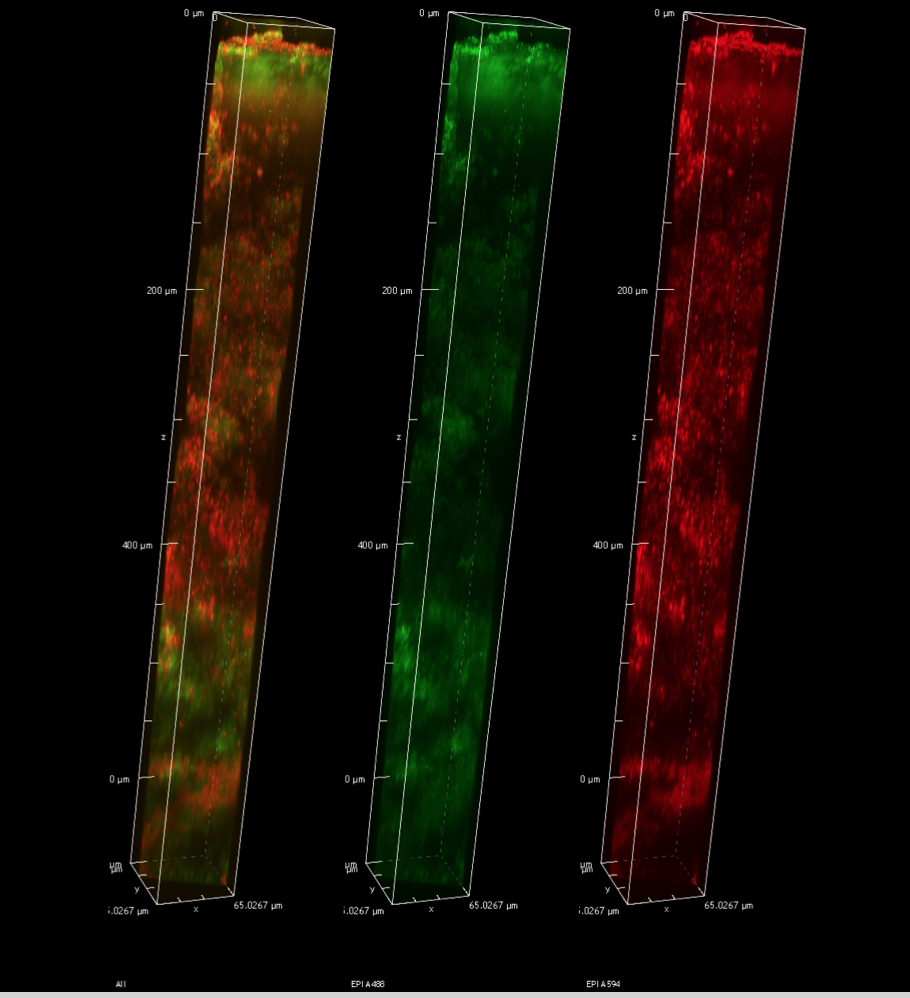
**

**PI**

**Syto-9**

**Merged**

**S2 Video. Multiphoton fluorescence 3D rendering of 9-day-old plaque biofilm along the dentin-composite interface up to 1 mm in depth (D-GL13-K treated).** Right channel (PI red=dead bacteria); central channel (Syto-9 green=live bacteria); left channel (red+green=merged live and dead bacteria).

**
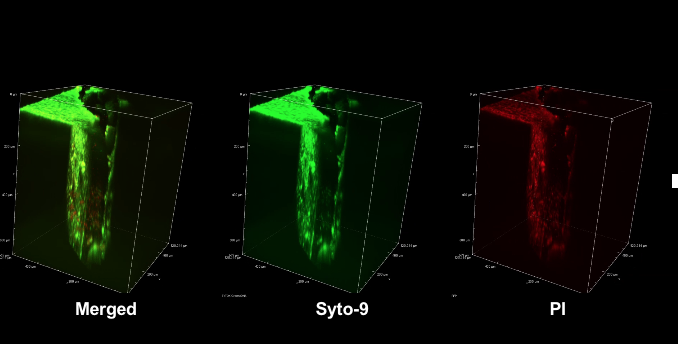
**

**S3 Video. Multiphoton fluorescence 3D rendering of the 18-day-old plaque biofilm at the interfacial gap between dentin and composite restoration (non-coated control).** Right channel (PI red=dead bacteria); central channel (Syto-9 green=live bacteria); left channel (red+green=merged live and dead bacteria).

**
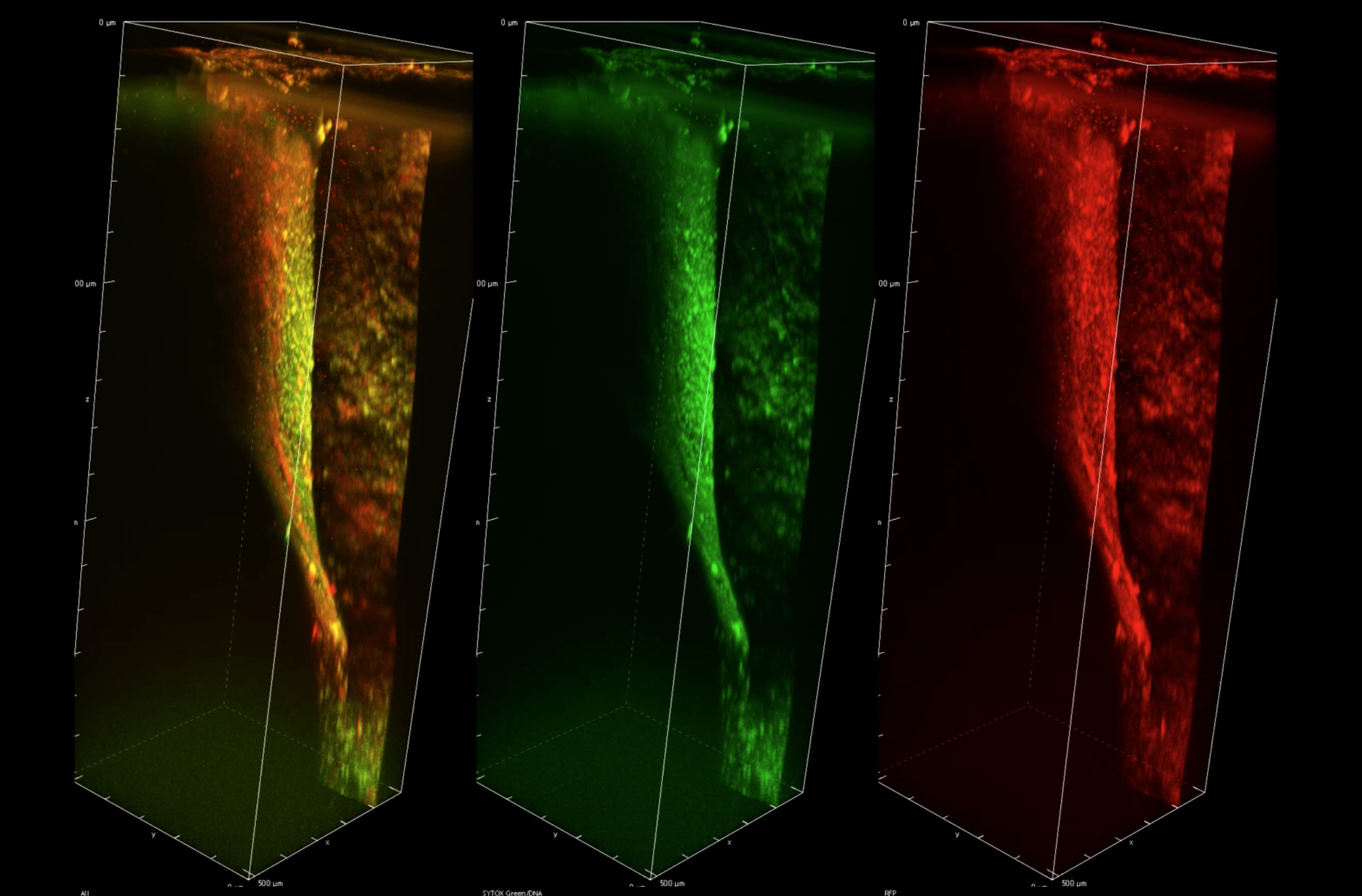
**

PI

Syto-9

Merged

**S4 Video. Multiphoton fluorescence 3D rendering of the 18-day-old plaque biofilm at the interfacial gap between dentin (straight surface) and composite (curved surface) restoration (D-GL13K-treated).** Right channel (PI red=dead bacteria); central channel (Syto-9 green=live bacteria); left channel (red+green=merged live and dead bacteria).

**S5 Fig. Effect of PMA treatment on dental plaque biofilms detached from AAMPs-coated HA discs using 16s rRNA gene next generation sequencing.** Principal coordinates plots show the distance and relatedness between taxonomic data of PMA-treated and non-PMA-treated samples (left panel). Alpha diversity plots show the genus-level change in bacterial richness (number of bacterial taxa) and bacterial evenness (relative abundance of bacterial taxa) with and without PMA treatment. Shannon index (top right panel) is sensitive to bacterial richness, Simpson index (middle right panel) is sensitive to bacterial evenness, InvSimpson (bottom right panel) is the inverse of Simpson index diversity estimator. PMA: propidium monoazide; AAMPs: amphipathic antimicrobial peptides; HA: hydroxyapatite; rRNA: ribosomal RNA.

**S6 Fig. Impact of D-GL13K-coated HA discs on the co-aggregation of plaque cultures.** Merged images of live/dead viability assay for 48h plaque cultures in proximity with etched-HA discs without (top) and with D-GL13K coatings (bottom).

**References**

1. Chen X, Hirt H, Li Y, Gorr SU, Aparicio C. Antimicrobial GL13K peptide coatings killed and ruptured the wall of Streptococcus gordonii and prevented formation and growth of biofilms. PLoS One. 2014;9(11):e111579. doi: 10.1371/journal.pone.0111579. PubMed PMID: 25372402; PubMed Central PMCID: PMC4221044.

2. de la Fuente-Nunez C, Reffuveille F, Haney EF, Straus SK, Hancock RE. Broad-spectrum anti-biofilm peptide that targets a cellular stress response. PLoS Pathog. 2014;10(5):e1004152. doi: 10.1371/journal.ppat.1004152. PubMed PMID: 24852171; PubMed Central PMCID: PMC4031209.

3. de la Fuente-Nunez C, Reffuveille F, Mansour SC, Reckseidler-Zenteno SL, Hernandez D, Brackman G, et al. D-enantiomeric peptides that eradicate wild-type and multidrug-resistant biofilms and protect against lethal Pseudomonas aeruginosa infections. Chem Biol. 2015;22(2):196-205. Epub 2015/02/24. doi: 10.1016/j.chembiol.2015.01.002. PubMed PMID: 25699603; PubMed Central PMCID: PMCPMC4362967.

4. Hirt H, Gorr SU. Antimicrobial peptide GL13K is effective in reducing biofilms of Pseudomonas aeruginosa. Antimicrob Agents Chemother. 2013;57(10):4903-10. Epub 2013/08/07. doi: 10.1128/AAC.00311-13. PubMed PMID: 23917321; PubMed Central PMCID: PMCPMC3811403.

5. Hirt H, Hall JW, Larson E, Gorr SU. A D-enantiomer of the antimicrobial peptide GL13K evades antimicrobial resistance in the Gram positive bacteria Enterococcus faecalis and Streptococcus gordonii. PLoS One. 2018;13(3):e0194900. Epub 2018/03/23. doi: 10.1371/journal.pone.0194900. PubMed PMID: 29566082; PubMed Central PMCID: PMCPMC5864073.

6. Nibbering PH, Ravensbergen E, Welling MM, van Berkel LA, van Berkel PH, Pauwels EK, et al. Human lactoferrin and peptides derived from its N terminus are highly effective against infections with antibiotic-resistant bacteria. Infect Immun. 2001;69(3):1469-76. Epub 2001/02/17. doi: 10.1128/IAI.69.3.1469-1476.2001. PubMed PMID: 11179314; PubMed Central PMCID: PMCPMC98043.

7. Mermillod-Blondin F, Fauvet G, Chalamet A, Châtelliers M. A Comparison of Two Ultrasonic Methods for Detaching Biofilms from Natural Substrata. Internat Rev Hydrobiol. 2001;86(3):349–60.
